# Switching from zoledronic acid to denosumab increases the risk for developing medication-related osteonecrosis of the jaw in patients with bone metastases

**DOI:** 10.1007/s00280-021-04262-w

**Published:** 2021-03-31

**Authors:** Hiroaki Ikesue, Kohei Doi, Mayu Morimoto, Masaki Hirabatake, Nobuyuki Muroi, Shinsuke Yamamoto, Toshihiko Takenobu, Tohru Hashida

**Affiliations:** 1 Department of Pharmacy, Kobe City Medical Center General Hospital, 2-1-1 Minatojima-Minamimachi, Chuo-ku, Kobe, Hyogo 650-0047 Japan; 2 Department of Oral and Maxillofacial Surgery, Kobe City Medical Center General Hospital, Kobe, Japan

**Keywords:** Denosumab, Zoledronic acid, Osteonecrosis of the jaw, Risk factor

## Abstract

**Purpose:**

Switch from zoledronic acid (ZA) to denosumab may increase the risk of medication-related osteonecrosis of the jaw (MRONJ) owing to the additive effect of denosumab on the jawbone and residual ZA activities. We evaluated the risk of developing MRONJ in patients who received ZA, denosumab, or ZA-to-denosumab for the treatment of bone metastases.

**Methods:**

The medical charts of patients with cancer who received denosumab or ZA for bone metastases were retrospectively reviewed. Patients who did not undergo a dental examination at baseline were excluded. Primary endpoint was the evaluation of the risk of developing MRONJ in the ZA-to-denosumab group. Secondary endpoints were probability of MRONJ and the relationship between risk factors and the time to the development of MRONJ.

**Results:**

Among the 795 patients included in this study, 65 (8.2%) developed MRONJ. The incidence of MRONJ was significantly higher in the ZA-to-denosumab group than in the ZA group [7/43 (16.3%) vs. 19/350 (5.4%), *p* = 0.007]. Multivariate Cox proportional hazards regression analysis revealed that denosumab treatment [hazard ratio (HR), 2.41; 95% confidence interval (CI), 1.37–4.39; *p* = 0.002], ZA-to-denosumab treatment (HR, 4.36; 95% CI, 1.63–10.54, *p* = 0.005), tooth extraction after starting ZA or denosumab (HR, 4.86; 95% CI, 2.75–8.36; *p* < 0.001), and concomitant use of antiangiogenic agents (HR, 1.78; 95% CI, 1.06–2.96; *p* = 0.030) were significant risk factors for MRONJ.

**Conclusion:**

Our results suggest that switching from ZA to denosumab significantly increases the risk for developing MRONJ in patients with bone metastases.

**Supplementary Information:**

The online version contains supplementary material available at 10.1007/s00280-021-04262-w.

## Introduction

Bone metastases are common in advanced cancers, resulting in clinically important complications, such as cancer-related pain, fractures, spinal cord compression, and hypercalcemia [1]. Skeletal-related events (SREs) remarkably decrease the quality of life of patients with bone metastasis. The effectiveness of bone-modifying agents (BMAs), such as zoledronic acid (ZA) and denosumab, in the treatment of bone metastases has been established. The results of randomized controlled trials comparing denosumab and ZA for the prevention of SREs in metastatic bone diseases have shown that denosumab is superior in cases of breast [2] and prostate cancer [3] and not inferior in cases of solid tumors and multiple myeloma [4, 5]. In addition, side effects, such as acute kidney injury, sometimes require the discontinuation of ZA. Thus, ZA has to be replaced with denosumab for some patients [6, 7].

Despite the effectiveness of BMAs, these medications can increase the risk of medication-related osteonecrosis of the jaw (MRONJ). MRONJ causes significant pain and reduces patient quality of life; therefore, multidisciplinary team care that enables appropriate monitoring and referral to a dental specialist for close follow-up and assessment of early stage MRONJ is recommended [8–10]. Several risk factors for MRONJ have been reported, including medication-, patient-, and oral health-related risk factors [8–10]. However, the risk of MRONJ in this patient population has not been fully evaluated. Both ZA and denosumab have been associated with MRONJ, but their pharmacological mechanisms are completely different. ZA has a high affinity for bone hydroxyapatite and specifically inhibits osteoclastic bone resorption and is therefore used for the treatment of bone metastases [11]. In contrast, denosumab is a fully humanized monoclonal antibody with high affinity and specificity for the nuclear factor-kappa B (NFκB) ligand RANKL. The effect of ZA on bone is long-lasting, whereas that of denosumab is temporary. We hypothesized that the risk of MRONJ may additively increase after switching from ZA to denosumab. An article had shown that switching from ZA-to-denosumab was one of the risk factors for developing MRONJ by logistic regression analysis, but not shown that switching increases risk directly [12].

In this study, therefore, we evaluated whether switching from ZA increase risk for developing MRONJ in cancer patients with bone metastases, comparing it to that in patients who received ZA/denosumab alone.

## Materials and methods

We retrospectively reviewed the medical records of patients with cancer who received denosumab and/or ZA for the treatment of bone metastases after dental examinations by dentists between Jul 2011 and Oct 2019.

### Study design, setting, and patient population

This study was conducted in accordance with the Declaration of Helsinki. The study protocol was approved by the Ethics Committee of the Kobe City Medical Center General Hospital (approval number: zn171010). Patients were eligible if they were ≥ 20 years of age, diagnosed with solid tumors or multiple myeloma, had at least one bone metastasis or osteolytic lesion, and received denosumab and/or ZA treatment at Kobe City Medical Center General Hospital between Jul 1, 2011 and Oct 31, 2019. The exclusion criteria were as follows: no dental examination before the initiation of denosumab or ZA treatment, use of ZA for the treatment of hypercalcemia, lack of follow-up for at least 1 month after the treatment, received denosumab followed by ZA, or history of radiation therapy of the jaws.

### Treatment procedure for bone metastases

Following dental examination, when needed, patients underwent dental procedures (including tooth extraction) to minimize the risk of developing MRONJ before the initiation of BMAs. All patients were subcutaneously administered 120 mg denosumab every 4 weeks or 4 mg ZA intravenously every 3 to 4 weeks. Patients with impaired kidney function (creatinine clearance of ≤ 60 mL/min) were given a manufacturer-recommended reduced dose of ZA (3–3.5 mg), according to the same administration schedule as that for patients with normal kidney function. We divided the study subjects into three groups as follows: patients who received only ZA (ZA group), only denosumab (denosumab group), and ZA followed by denosumab (ZA-to-denosumab group).

### Data collection and assessment

All data were collected from the electronic medical record system. We evaluated information regarding sex, age, weight, type of cancer, comorbidities, tooth extraction before and after starting BMA treatments, concomitant medications, type of BMAs, number of treatment courses, and outcomes of treatment for MRONJ. To reduce the potential bias in evaluating patient and treatment characteristics associated with the development of MRONJ, we limited the study participants to those examined by dentists before starting BMA treatments because poor oral health status has been reported as a significant risk factor for developing MRONJ [8–10]. Furthermore, all patients were recommended to visit dental clinics routinely after BMA initiation. If the patients were considering invasive dental procedures, including tooth extraction, after the initiation of treatment with BMAs, they were asked to consult with dentists in our hospital. After the initiation of BMA treatment, patients who complained of dental symptoms, such as pain or oral discomfort, consulted with a dentist following the attending physician’s request. Tooth extraction was performed in unavoidable situations, including accidental root fracture or acute exacerbation of periodontal disease. MRONJ was diagnosed by dentists in our hospital based on clinical and radiographic findings, according to the criteria stated in the American Association of Oral and Maxillofacial Surgeons (AAOMS) position paper [13], and the cutoff date for diagnosing MRONJ was Dec 31, 2019. The primary endpoint was the evaluation of the risk of developing MRONJ in the ZA-to-denosumab group, whereas secondary endpoints included the probability of MRONJ and the relationship between risk factors and the time to the development of MRONJ.

### Statistical analysis

Categorical data are presented as numbers (percentage) and were compared between groups using the Chi-square test or Fisher’s exact test, as appropriate. Continuous data are presented as medians (interquartile ranges), and the Mann–Whitney *U* test was used to compare the groups. Univariate and multivariate Cox proportional hazards regression models were used to identify the risk factors for MRONJ. Variables with a *p *value < 0.05 in the univariate analysis were evaluated as potential covariates in the multivariate analysis. The time to the development of MRONJ was determined using the Kaplan–Meier method with the log-rank test. All statistical analyses were performed using JMP 13.0.0 (SAS Institute Inc., Cary NC, USA). A *p* value < 0.05 was considered statistically significant. For comparisons of the incidences of MRONJ between anti-resorptive treatment groups, the Bonferroni correction was applied to determine the level of significance for each group (*p* < 0.0167).

## Results

### Patient characteristics

Between Jul 2011 and Oct 2019, 1192 adult patients with cancer bone metastases were treated with denosumab and/or ZA. Among these, 397 patients were excluded because they received ZA for the treatment of hypercalcemia (*n* = 163), did not undergo dental examinations before the initiation of treatment with denosumab or ZA (*n* = 148), could not be followed up for at least 1 month after treatment (*n* = 82), or switched from denosumab to ZA (*n* = 4). The remaining 795 patients (350 in the ZA group, 402 in the denosumab group, and 43 in the ZA-to-denosumab group) were the study subjects. In the ZA-to-denosumab group, the median [interquartile range (IQR)] number of infusions of ZA was 8 (2–17), and 65 patients (8.2%) developed MRONJ. Patient characteristics are shown in Table 1. In the ZA-to-denosumab group, the median (IQR) number of ZA dosages tended to be higher in patients who developed MRONJ than in those who did not [13 (5–26) vs. 6 (2–16), *p* = 0.092]. Within this group, no factor was significantly different between the patients who developed MRONJ and those who did not.

**Table 1:** Patient characteristics

Characteristics	ZA (*n* = 350)	Denosumab (*n* = 402)	ZA-to-denosumab (*n* = 43)
Male sex, *n* (%)	175 (50.0%)	222 (55.2%)	13 (30.2%)
Age (years), median (IQR)	68 (60–75)	69 (61–75)	61 (53–68)
Type of disease, *n* (%)			
Lung cancer	85 (24.3%)	183 (45.5%)	13 (30.2%)
Breast cancer	43 (12.3%)	86 (21.4%)	20 (46.5%)
Multiple myeloma	120 (34.3%)	6 (1.5%)	3 (7.0%)
Prostate cancer	34 (9.7%)	83 (20.7%)	3 (7.0%)
Others	68 (19.4%)	44 (11.0%)	4 (9.3%)
Tooth extraction before starting BMAs, *n* (%)	74 (21.1%)	92 (22.9%)	8 (18.6%)
Comorbid with diabetes, *n* (%)	62 (17.7%)	74 (18.4%)	7 (16.3%)
Concomitant medication, *n* (%)			
Antiangiogenic agents ^a^	61 (17.4%)	102 (25.4%)	23 (53.5%)
Corticosteroids	31 (8.9%)	58 (14.4%)	6 (14.0%)
Tooth extraction after starting BMAs, *n* (%)	30 (8.6%)	36 (9.0%)	4 (9.3%)
Number of treatment courses, median (IQR)			
Zoledronic acid	6 (3–16)	0	8 (2–17)
Denosumab	0	8 (3–17)	8 (3–19)

For continuous values, data are presented as the median [interquartile range (IQR)]

*BMA* bone-modifying agent, *MRONJ* medication-related osteonecrosis of the jaw, *ZA* zoledronic acid

^a^Includes axitinib, bevacizumab, everolimus, lenvatinib, pazopanib, ramucirumab, regorafenib, sorafenib, sunitinib, and temsirolimus

### Risk factors for MRONJ

**Fig. 1:**
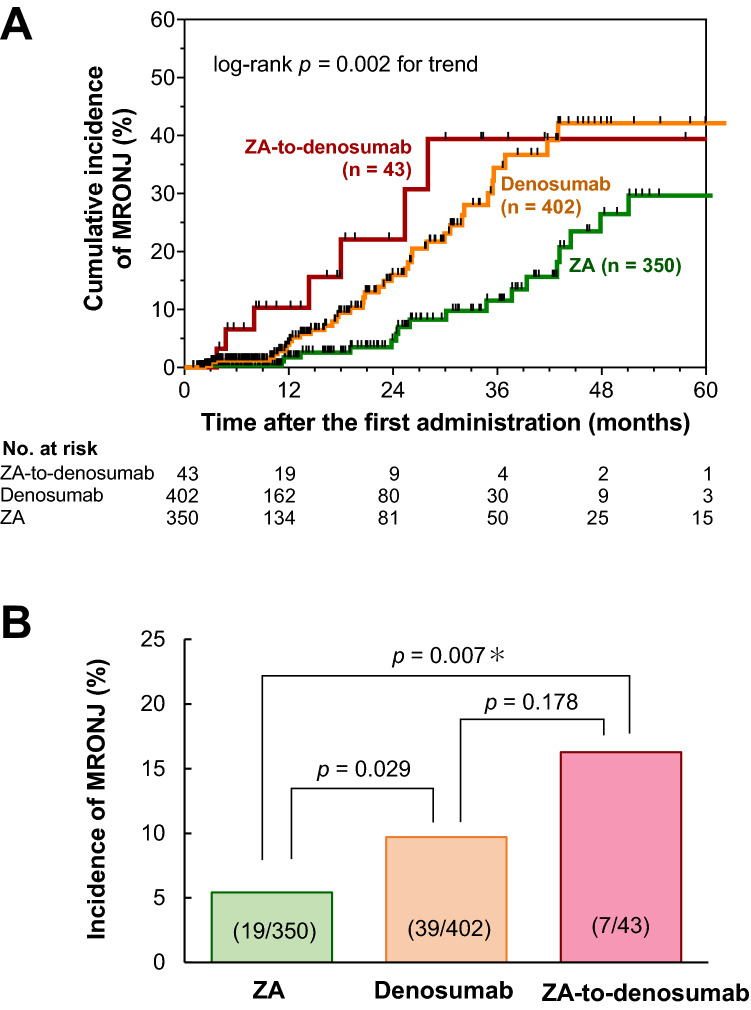
Incidences of medication-related osteonecrosis of the jaw in patients receiving denosumab or zoledronic acid for bone metastases. Kaplan–Meier curves of cumulative incidences of MRONJ (**a**) and the incidences of MRONJ (**b**) in patients of the ZA alone (*n *= 350), denosumab alone (*n* = 402), and ZA-to-denosumab (*n* = 43) groups are shown. In the ZA-to-denosumab group, patients received a median (IQR) of 8 (2–17) ZA infusions before the first dose of denosumab. *Statistical significance was considered at a *p* value < 0.0167 for the Chi-square test (the criteria for significance were adjusted using Bonferroni correction). *IQR* interquartile range, *MRONJ* medication-related osteonecrosis of the jaw, *ZA* zoledronic acid

Univariate analysis showed that treatment with denosumab [hazard ratio (HR), 2.32; 95% confidence interval (CI), 1.34–4.17; *p* = 0.002], sequential treatment with ZA and denosumab (HR, 3.63; 95% CI, 1.41–8.36; *p* = 0.010), tooth extraction before starting BMAs (HR, 1.94; 95% CI, 1.16–3.19; *p* = 0.012), concomitant use of antiangiogenic agents (HR, 2.24; 95% CI, 1.35–3.67; *p* = 0.002), and tooth extraction after starting BMAs (HR, 4.38; 95% CI, 2.55–7.30; *p* < 0.001) were significantly associated with the development of MRONJ in patients with cancer who received treatment with BMAs (supplementary Table S1). Subsequent multivariate Cox proportional hazards regression analysis also showed that denosumab treatment (HR, 2.41; 95% CI, 1.37–4.39; *p* = 0.002), sequential treatment with ZA and denosumab (HR, 4.36; 95% CI, 1.63–10.54; *p* = 0.005), tooth extraction after starting BMAs (HR, 4.86; 95% CI, 2.75–8.36; *p* < 0.001), and concomitant use of antiangiogenic agents (HR, 1.78; 95% CI, 1.06–2.96; *p* = 0.030) were significantly associated with a risk of developing MRONJ in patients with cancer who received treatment with BMAs. To further explore the relationship between these risk factors and MRONJ development, we analyzed the time to the onset of MRONJ using Kaplan–Meier analysis (Fig. 1a). The cumulative incidence of MRONJ was significantly different among the three groups in this study (*p* = 0.002 for trend, log-rank test). The incidence of MRONJ in the ZA-to-denosumab group was significantly higher than that in the ZA group (16.3 vs. 5.4%, *p* = 0.007), whereas it was not significantly different from that in the denosumab group (9.7%) after Bonferroni correction (Fig. 1b).

## Discussion

In the present study, we showed for the first time that among all patients who received dental examinations before BMA treatment for bone metastases, ZA-to-denosumab treatment significantly increased the risk of developing MRONJ, when compared to that with ZA. Concomitant use of antiangiogenic agents and tooth extraction after starting BMA treatment were also significant risk factors. Our study results clearly showed that the highest incidence of MRONJ was observed in the ZA-to-denosumab group. This information is important for minimizing the toxicity of anti-resorptive treatments in cancer patients with bone metastasis, since BMA treatment needs to be switched from ZA to denosumab in some patients, such as with skeletal disease progression or ZA-induced acute kidney injury [6, 7]. Bisphosphonates, including ZA, are known to have a high affinity for hydroxyapatite of bone [11], leading to prolonged drug action and excessive toxic effects. Therefore, in patients who switched from ZA to denosumab treatment, the additive effects of denosumab on the jawbone and the residual effect of ZA may increase the risk of MRONJ. Higuchi et al. conducted a single center, retrospective chart review and revealed that switching from ZA to denosumab is one of the risk factors in logistic regression analysis [12]. However, that study did not directly show the risk itself by comparing the switching group to ZA/denosumab alone group. In contrast, in an extended observation of two phase III trials, the incidence of MRONJ did not increase in patients who received ZA followed by denosumab [14]. Our study, for the first time, fully evaluated the risk of developing MRONJ in patients who received ZA followed by denosumab, comparing it to that in patients who received ZA alone, in a clinical practice setting. As part of a comprehensive pharmacovigilance plan, a prospective, post-marketing drug surveillance of cancer patients with bone metastases receiving antiresorptive therapies is ongoing in Denmark, Sweden, and Norway [15]. The observational period of this surveillance is up to 5 years, and the results will be reported for three treatment cohorts as follows: denosumab-naïve patients, ZA-naïve patients, and patients who switch from bisphosphonate treatment to denosumab. The results of the study will further clarify the relationship between the characteristics of BMAs and their effects on MRONJ.

The reported incidence of MRONJ is 1–17% [2–5, 12, 14, 16–19]. The incidence of MRONJ in the present study was within this range for ZA (5.4%), denosumab (9.7%), and ZA-to-denosumab (16.3%) groups. Importantly, none of the patients in the ZA-to-denosumab group developed MRONJ while receiving ZA, but seven of these 43 patients developed MRONJ after switching to denosumab. Our multivariate analysis revealed that patients in both the denosumab and ZA-to-denosumab groups had a significantly higher risk of developing MRONJ than those treated with ZA. In contrast, previous randomized controlled trials showed that the incidence of MRONJ in patients treated with denosumab was not significantly different from that in patients treated with ZA, although it tended to be higher [3, 17, 18]. This discordance might be attributed to the scheduled periodic dental examinations (e.g., at baseline and every 6 months thereafter) in previous randomized clinical trials, which decreased the risk of developing MRONJ [2–4, 17]. In fact, a recent meta-analysis of eight randomized controlled trials found a remarkably higher risk of developing MRONJ in patients treated with denosumab than in those treated with ZA [20]. The higher incidence of denosumab-associated ONJ seems to reflect the superior effect of denosumab in preventing skeletal-related events, compared to that with ZA [2, 3].

The median number of infusions of ZA was 8. Since most patients received ZA every 4 weeks in our study, patients received ZA treatment for approximately 8 months. Subsequently, BMAs were usually switched to denosumab treatment, and the cumulative incidence of MRONJ in the ZA-to-denosumab group was higher than that in the denosumab or ZA alone groups from 4 months after the first administration of denosumab. Therefore, the difference was evident at 12 months from the first administration of ZA, which seems early. We speculate that a relative lack of awareness of dental follow-up in clinical practice compared to that with prospective intervention studies might have influenced this marked difference in the cumulative incidence of MRONJ.

The other independent risk factors for developing MRONJ in this study were concomitant use of antiangiogenic agents and tooth extraction after starting BMAs, which were consistent with the findings of previous reports [10, 16, 19]. Our result may support the notion that tooth extraction before starting BMAs is a useful prophylactic intervention to reduce the risk of developing MRONJ. However, because tooth extraction before starting BMAs significantly increased the risk of developing MRONJ in the univariate analysis, early dental consultation should be considered after patients are diagnosed with cancer.

This study has some limitations. First, oral health status, such as periodontal disease, dental prosthesis, dental implants, and periodontal surgeries, was not fully investigated in our retrospective observational study design. To reduce the effect of these factors, we limited the study participants to those examined by dentists before starting BMA treatments. Second, we did not evaluate the effect of other risk factors, such as denture use and tobacco use [8–10]. Despite our best attempt to obtain clinical information, we were not able to collect all these data with this retrospective study design. To our knowledge, however, these missing data should have similar impacts among the groups. Lastly, the IQR of the number of infusions of ZA in the ZA-to-denosumab group varied from 2 to 17, indicating that patients with various backgrounds were included in this group. Despite these limitations, this real-world observational study demonstrated that the risk of developing MRONJ was significantly higher in patients with advanced cancer treated with ZA followed by denosumab. In conclusion, the results of this study suggest that switching from ZA to denosumab significantly increases the risk of developing MRONJ in patients with bone metastases.

## Supplementary Information

Below is the link to the electronic supplementary material.

Supplementary file1 (PDF 85 KB)
