# Supplementary material for: Switching from zoledronic acid to denosumab increases the risk for developing medication-related osteonecrosis of the jaw in patients with bone metastases
Source: Cancer Chemother Pharmacol. 2021 Mar 31;87(6):871–7. doi: 10.1007/s00280-021-04262-w (PMC8110486; doi:10.1007/s00280-021-04262-w)
Supplement: Supplementary file 1 — Supplementary file1 (PDF 85 KB) [file 280_2021_4262_MOESM1_ESM.pdf]

Supplementary Table

Title: Switching from zoledronic acid to denosumab increases the risk for developing medication-related osteonecrosis of the jaw in patients with bone metastases

Authors: Ikesue H, Doi K, Morimoto M, Hirabatake M, Muroi N, Yamamoto S, Takenobu T, and Hashida T.

Supplementary Table S1. Univariate and multivariate analyses of risk factors for medication-related osteonecrosis of the jaw in patients who received denosumab or zoledronic acid for bone metastases

Variables	Univariate analysis		Multivariate analysis	
	HR (95% CI)	*P*-value	HR (95% CI)	*P*-value
Bone-modifying agents				
ZA (control)	1.00	–	1.00	–
Denosumab	2.32 (1.34–4.17)	0.002	2.41 (1.37–4.39)	0.002
ZA followed by denosumab	3.63 (1.41–8.36)	0.010	4.36 (1.63–10.54)	0.005
Tooth extraction after starting BMAs	4.38 (2.55–7.30)	<0.001	4.86 (2.75–8.36)	<0.001
Concomitant use of antiangiogenic agents [a]	2.24 (1.35–3.67)	0.002	1.78 (1.06–2.96)	0.030
Tooth extraction before starting BMAs	1.94 (1.16–3.19)	0.012	1.36 (0.79–2.28)	0.261
Concomitant use of corticosteroids	0.99 (0.53–1.75)	0.983	N/A	
Age (years)	1.01 (0.99–1.03)	0.503	N/A	
Male sex	0.98 (0.60–1.61)	0.949	N/A	
Diabetes	0.49 (0.12–1.40)	0.201	N/A	

BMA, bone modifying agent; CI, confidence interval; HR, hazard ratio; ZA, zoledronic acid.
N/A indicates that the covariate was not included in the model because it was not significant in univariate analyses.
[a] Includes axitinib, bevacizumab, everolimus, lenvatinib, pazopanib, ramucirumab, regorafenib, sorafenib, sunitinib, and temsirolimus
